# DEVELOPMENT OF A BEHAVIORAL FRAMEWORK FOR DEMENTIA CARE PARTNERS’ FALL RISK MANAGEMENT

**DOI:** 10.1093/geroni/igac059.2017

**Published:** 2022-12-20

**Authors:** Yuanjin Zhou, Clara Berridge, Nancy Hooyman, Tatiana Sadak, Tracy Mroz, Elizabeth Phelan

**Affiliations:** University of Texas at Austin, Austin, Texas, United States; University of Washington, Seattle, Washington, United States; University of Washington, Seattle, Washington, United States; University of Washington, Seattle, Washington, United States; University of Washington, Seattle, Washington, United States; University of Washington, Seattle, Washington, United States

## Abstract

Although older adults living with dementia (OLWD) are at high risk for falls, few strategies that effectively reduce falls among OLWD have been identified. Dementia care partners may have a critical role in fall risk management (FRM). However, little is known in what ways care partners behave that may be relevant to FRM and how to effectively engage them in FRM. Semi-structured, in-depth interviews were conducted with 14 primary care partners (Age: 48-87; 79% women; 50% spouses/partners; 64% college-educated; 21% people of color) of community-dwelling OLWD to ascertain their own behaviors and those of care partners who were secondary in the caring role. The analysis of interview data suggested a novel behavioral framework consisting of eight domains of FRM behaviors adopted across four stages. The domains were 1. functional mobility assistance, 2. assessing and addressing health conditions, 3. health promotion support, 4. safety supervision, 5. modification of the physical environment, 6. receiving, seeking, and coordinating care, 7. learning, and 8. self-adjustment. Four stages of FRM included 1. supporting before dementia onset, 2. preventing falls, 3. preparing to respond to falls, and 4. responding to falls. FRM behaviors varied by the care partners’ caring role. Primary care partners engaged in behaviors from all eight domains. Secondary care partners were reported to assist in health promotion support, safety supervision, modification of the physical environment, and receiving, seeking, and coordinating care. This multi-domain and multi-stage framework will inform intervention development to engage care partners in managing fall risk for community-dwelling OLWD.

